# Combined Chronotherapy for Poor Sleep Following Acute Coronary Syndrome: A Pilot Randomized Trial

**DOI:** 10.5334/jcr.250

**Published:** 2025-02-25

**Authors:** Miguel Mendieta, Robin Cumella, Nakesha Fray, David Lopez-Veneros, David Hiti, Christina Franqui, Consuelo D’Agostino, Ian M. Kronish, Ari Shechter

**Affiliations:** 1 Center for Behavioral Cardiovascular Health, Department of Medicine, Columbia University Irving Medical Center, US; 2 School of Nursing, Columbia University Irving Medical Center, US; 3 Department of Emergency Medicine, Columbia University Irving Medical Center, US; 4 Columbia Interventional Cardiovascular Care, New York-Presbyterian/Columbia University Irving Medical Center, US

**Keywords:** sleep, insomnia, bright light therapy, blue light, chronotherapy, cardiac

## Abstract

Sleep disturbance is common following acute coronary syndrome (ACS) and may contribute to a worse prognosis. We describe the rationale, design, and findings of a pilot and feasibility randomized clinical trial (RCT) of an intervention to improve post-ACS sleep. Fifteen post-ACS patients with insomnia and/or short sleep were randomized (2:1 allocation) to combined chronotherapy (CC) plus sleep hygiene education (SHE) or SHE control. CC was bright light therapy (BLT) delivered by a wearable light visor for 30 minutes after awakening and short-wavelength light avoidance using blue-light blocking (BLB) glasses in the evening from 8:00pm to bedtime daily for 4 weeks. Primary outcomes were feasibility, acceptability, appropriateness, usability, and adherence. Secondary outcomes were insomnia symptoms, sleep quality, and sleep duration. Two patients in the CC intervention group dropped before initiating study procedures. Completion of study procedures in remaining patients was high in the CC and control groups (88% and 100%, respectively). Self-reported adherence to CC (i.e., using BLT and BLB on ≥50% of days) was high (88% and 100%, respectively). The proportion of CC patients who perceived the intervention as feasible (71%) and usable (100%) was high, though fewer CC patients rated the intervention as acceptable (57%) and appropriate to improve sleep (29%). Improvements in insomnia symptoms and self-reported sleep quality and duration were seen in response to the CC intervention (71%). Post-ACS patients with sleep disturbance had high adherence to this chronotherapeutic intervention, and most viewed it as feasible and usable. This intervention should be tested in a larger RCT to determine efficacy to improve sleep.

**Trial Registration:**

ClinicalTrials.gov number NCT05299723

**Date of registration:**

March 29, 2022

**URL of trial registry record:**

https://classic.clinicaltrials.gov/ct2/show/NCT05299723

## Introduction

Evidence indicates a causal (and potentially bidirectional) relationship between sleep disturbance and cardiovascular disease (CVD) risk [1,2]. This sleep-CVD link has been shown for short sleep duration [3] and insomnia [4]. Several mechanisms have been proposed for this association. For example, short sleep duration is experimentally shown to raise blood pressure, attenuate endothelial function, and increase sympathetic activation [5]. Similarly, insomnia is often characterized by psycho-physiologic stress and hyperarousal [6] and associated dysregulation of the autonomic nervous system (e.g., increased blood pressure and heart rate, decreased parasympathetic tone), hypothalamic-pituitary-adrenal axis function (e.g., increased norepinephrine and evening cortisol), and systemic inflammation [7,8]. The critical role of sleep in CVD prevention is being increasingly recognized. In 2022, the American Heart Association added sleep as an eighth factor (joining smoking, obesity, cholesterol levels, glucose levels, blood pressure, physical activity, and diet) used to define optimal cardiovascular health (“Life’s Essential 8”) [9].

In addition to contributing to incident CVD, sleep disturbances are common following acute cardiac events. The prevalence of self-reported short sleep duration in survivors of acute coronary syndrome (ACS; i.e., myocardial infarction, unstable angina) was found to be ~10% to up to 50% based on different definitions of short sleep (e.g., <6 hours or <7 hours per night) [10,11]. In those studies, short (vs. not short) sleep duration was associated with an increased risk of recurrent cardiac event and/or mortality of ~50% across 1-year [10] and ~30% over a median 2.5-year follow-up [11]. The prevalence of insomnia in patients who experienced ACS was also high. Approximately ~50% of patients indicated clinically relevant sleep disturbance (i.e., insomnia symptoms based on a score of 5 or higher on the Jenkins Sleep Scale), which persisted across the first year following ACS [12]. This is relevant since insomnia symptoms have been prospectively associated with recurrent coronary events and/or mortality following ACS [13,14,15]. Sleep disturbance, including short sleep duration and/or insomnia, may play a role in recurrent cardiac event risk and may be a secondary prevention target to improve prognosis in these patients.

A few studies have tested interventions that target sleep disturbances in cardiac patients. These studies show that administering cognitive behavioral therapy for insomnia (CBT-I), considered the first-line non-pharmacologic treatment, is both feasible and efficacious in patients with heart failure and insomnia [16]. To our knowledge, no studies have examined the impact of sleep interventions on long-term cardiac outcomes. Studies have demonstrated a reduction in blood pressure following CBT-I in patients with hypertension and insomnia [17] and reductions in blood pressure following a behavioral sleep extension intervention in patients with short sleep duration and prehypertension or stage 1 hypertension [18,19]. These suggest that interventions to improve sleep disturbance following ACS may reduce secondary risk in these patients.

Established interventions for sleep disturbance that have been developed and implemented are not without limitations. For example, barriers to CBT-I implementation include limited access and low referral to CBT-I providers, high costs, time and social constraints for patients, and long wait times [20]. The use of exogenous melatonin as a sleep aid has risen drastically over the past 20 years [21], yet the safety and efficacy of its use remain uncertain [22]. Pharmacologic approaches to sleep disturbance, including use of hypnotics, may have adverse side effects and confer increased safety risks (e.g., falls) [23]. This may be of particular concern in ACS patients who are generally older and prescribed multiple medications. Some evidence suggests that patients with insomnia, including those in in-patient settings, may prefer non-pharmacologic over pharmacologic approaches [24,25]. Altogether, there is the potential advantage of developing novel non-pharmacologic approaches to disturbed sleep.

The development and widespread use of artificial electric light has allowed humans to shape their light environment, although often with deleterious effects on sleep. A recent report found that nearly half of home environments had light levels in the evening that were bright enough to suppress melatonin by 50%, and these higher levels of evening light were associated with poorer nighttime sleep [26]. Moreover, exposure to light, particularly short-wavelength light within the blue portion of the visible spectrum (“blue light”), at night delays and dampens melatonin secretion [27] and induces neurophysiologic arousal [28]. These can disrupt sleep initiation and maintenance. This is critical since use of light-emitting devices before bedtime is ubiquitous, with over 90% of individuals reporting continued use within the hour before bedtime [29]. Light exposure from eReaders (i.e., tablets) [30] and light-emitting diode (LED) computer screens [31] enriched in short-wavelength light decreases sleepiness during the hours preceding bedtime [30,31], prolongs sleep onset [30], and worsens sleep quality [30,32]. Most computer, tablet, TV, and smartphone screens have LEDs with peak wavelength in the short-wavelength range [31]. Conversely, most individuals rarely have adequate exposure to bright and natural light during the daytime, including during the beneficial morning hours [33]. Low daytime light levels are associated with disturbed sleep, fatigue, lack of concentration, and depressed mood [34,35]. Thus, most people are paradoxically exposed to overly dim light levels during the daytime when bright light exposure is ideal for maintaining proper circadian synchronization while exposed to excessive light levels at night. In analyses that considered the total daily light exposure pattern, a larger evening light-to-daytime light intensity ratio (i.e., brighter nights and dimmer days) was associated with a disturbed circadian system [35]. This is likely to contribute to sleep and mood disturbances.

Chronotherapeutic or circadian rhythm-based treatment approaches can target exposure to environmental stimuli that influence the circadian system [36]. Environmental light is the strongest circadian time-setting (synchronizing) cue. Light-based therapies can help maintain proper circadian function, boost circadian amplitude, and ensure that the body’s biological rhythm is synchronized to the day-night solar cycle. Timed bright light exposure can improve sleep and related symptoms [36]. A meta-analysis found that bright light therapy (BLT) is effective in improving sleep problems [37]. In a crossover randomized trial, we previously observed that using blue-light blocking (BLB) lenses worn in frames before bedtime improved insomnia severity and increased sleep duration in individuals with insomnia symptoms [38]. Further, a meta-analysis on BLB techniques to improve sleep demonstrated the effectiveness of BLB at night for improving sleep quality ratings and increasing sleep duration, particularly in individuals with sleep problems [39].

**Figure 1:**
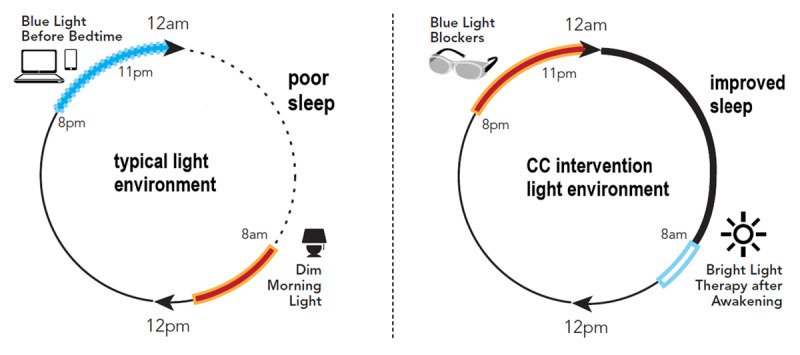
The typical 24-hour light environment, characterized by short-wavelength light exposure before bedtime and dim light in the morning, is shown on the left. The “combined chronotherapy” (CC) approach, including short-wavelength (“blue”) light blocking in the hours preceding bedtime and bright light exposure after awakening, is shown on the right.

Although research has been done to test the effects of BLT and BLB separately on sleep, little work to date has utilized them together in a “combined chronotherapy” (CC) approach. Further, to our knowledge, no light-based chronotherapeutic intervention has been developed specifically to improve sleep in cardiac patients. Accordingly, the logical next step in interventional development, according to the NIH Stage Model [40], is to develop the intervention by adapting existing interventions into a combined protocol and then test the feasibility of this intervention in ACS patients who are at high risk of adverse consequences from poor sleep (i.e., Stage IA/IB research). Here, we describe the pilot and feasibility testing of a CC intervention consisting of morning BLT and evening BLB plus sleep hygiene education (SHE), which aims to alleviate circadian desynchrony and improve sleep by augmenting beneficial (bright) daytime light and reducing sleep/circadian-disturbing short-wavelength light exposure at night following ACS (Figure 1).

## Materials and Methods

### Overview of Study Design

We conducted and report this study in accordance with the CONSORT extension for pilot RCTs (Supplementary Table). The SleepWell Study was a remotely administered interventional pilot study targeting disturbed sleep in patients who experienced a recent ACS event. The intervention consisted of a CC intervention administered over four weeks through a combination of BLT in the mornings (from a wearable light-emitting visor) and reduced exposure to short-wavelength light in the evenings (from orange-colored, BLB lenses worn in frames that can fit over prescription glasses). In addition, SHE was provided to patients via two brief educational videos as part of the intervention. SHE alone was provided to patients in the control group. The study was a parallel-arm RCT of this home-based CC + SHE intervention in 15 post-ACS patients to test the feasibility, acceptability, appropriateness, adherence, study/outcomes completion, and effects of the intervention on sleep. The Columbia University Irving Medical Center Institutional Review Board (IRB-AAAU0150) approved all study procedures, and all patients provided informed verbal consent.

### Study procedures

Table 1 presents a timeline for key activities in the trial, including screening, enrollment, and outcomes assessment time points. Following informed consent and screening, research staff enrolled patients and randomized them into treatment groups (see below). All patients completed two study visits across four weeks: (1) a baseline/pre-intervention session and (2) a post-intervention/study period session. All study sessions could have been completed via video teleconference, over the telephone, or in person, depending on patient preference. Once research staff established patient eligibility, each patient was sent study materials. These materials consisted of a sleep and device usage log sent via mail to the patient’s home, as well as links to two educational videos concentrating on sleep hygiene (SHE videos) sent via email. Patients assigned to the CC + SHE group were also provided intervention devices, including a light visor (Luminette 3 Light Therapy Glasses) and glasses with orange-colored short-wavelength blocking (blue-light-blocking, BLB) lenses (LowBlueLights Zzz Fitover Sleep glasses).

**Table 1:** Timeline of the trial.


	SCREENING SESSION	BASELINE SESSION	INTERVENTION STUDY PERIOD				POST-INTERVENTION FOLLOW-UP	

OUTCOMES		MONTH 0	WEEK 1	WEEK 2	WEEK 3	WEEK 4	MONTH 1	EXIT INTERVIEW

**Screening and enrollment**								

Informed verbal consent ^a^	◯							

Eligibility screening ^a^	◯							

Demographics ^a^	◯							

**Interventions**								

BLB & BLT ^b^			◯	◯	◯	◯		

**Assessments**								

ISI ^a^		◯					◯	

PSQI ^a^		◯					◯	

AIM ^b^							◯	

IAM ^b^							◯	

FIM ^b^							◯	

SUS ^b^							◯	

Intervention adherence ^b^			◯	◯	◯	◯		◯

Adverse events ^b^			◯	◯	◯	◯		


BLB: Blue light blocking; BLT: Bright light therapy; ISI: Insomnia Severity Index; PSQI: Pittsburgh Sleep Quality Index; AIM: Acceptability of Intervention Measure; IAM: Intervention Appropriateness Measure; FIM: Feasibility of Intervention Measure; SUS: System Usability Scale. ^a^ Conducted in all patients. ^b^ Conducted in intervention group patients.

Patients underwent the baseline/pre-intervention session after they confirmed via telephone that they received study materials and viewed the SHE videos. Patients completed questionnaires about their sleep (see Outcomes below). Once patients completed questionnaires, study staff reviewed all study documents, instructed patients on device use and answered any questions. Patients in the CC + SHE group began the intervention after completing the baseline/pre-intervention session and staff instructed patients to use the study devices daily for four weeks. All patients were asked to complete a daily sleep diary, logging their bed and wake times. CC + SHE group patients completed a sleep diary that also contained a device use log, in which they documented the times they used the BLT and BLB devices each day. All patients completed one brief weekly check-in by telephone with study staff for four weeks following the initiation of the intervention/study period. During weekly check-ins, research staff asked patients about the occurrence of any adverse events from the intervention and reported their responses from the sleep and device use log to research personnel. The fourth and final weekly check-in was considered the end of the intervention, at which point patients in the CC + SHE group were instructed to stop using the study devices. The post-intervention session was conducted approximately four weeks after completing the baseline/pre-intervention session and entailed the completion of the same questionnaires as baseline and, in the CC + SHE group, questionnaires pertaining to the patient’s perceptions of the intervention.

Compensation was provided to patients for completing all study components, including separate payments for completing the baseline/pre-intervention session, completing the post-intervention session, and returning the sleep diary/daily-use log and BLT visor. Compensation was not contingent upon completing any intervention component (so as not to confound feasibility measures). Patients were able to keep the BLB glasses.

### Patients

The inclusion and exclusion criteria for the study are described in Table 2. Inclusion criteria were: (1) 18 years of age or older; (2) provider/medical record-confirmed ACS; (3) ACS event occurred within the past three months; and (4) presence of insomnia symptoms based on the Insomnia Symptoms Questionnaire (“present” or “absent” based on meeting criteria) or short sleep duration defined as frequently (3–4 times per week) or always (5–7 times per week) experiencing 6 hours or less of sleep per night.

**Table 2:** Summary of eligibility criteria.


INCLUSION CRITERIA	EXCLUSION FOR CRITERIA

Age ≥ 18 years Confirmed ACS event within past 3 months Self-reported sleep disturbance at baseline: screening positive for chronic insomnia symptoms or by reporting habitual short sleep duration	Severe disabling chronic medical and/or psychiatric comorbidity Deemed unable to comply with the protocol by investigator or clinician Not able to speak, read and understand English or Spanish Unavailable for follow-up for reasons such as terminal illness and imminent plans to leave the United States Unreliable phone or e-mail access History of bipolar disorder or positive screen for bipolar disorder based on the Mood Disorder Questionnaire Diagnoses with eye disease (including glaucoma or retinopathy) or blindness Night shift work Taking anti-depressant or anti-anxiety medications Taking medications that increase sensitivity to light (by self-report)


ACS: Acute coronary syndrome.

Exclusion criteria were: (1) severe disabling chronic medical and/or psychiatric comorbidities determined on a case-by-case basis that prevented safe or adequate participation; (2) could not speak, write and read English or Spanish; (3) deemed unable to comply with the protocol (either self-reported or indicated during screening that they could not complete all requested tasks); including but not limited to, patients with a level of cognitive impairment indicative of dementia, patients with current alcohol or substance abuse, and patients with severe mental illness (e.g., schizophrenia); (4) unavailable for follow-up for reasons such as terminal illness or imminent plans to leave the United States; (5) lack of reliable phone or e-mail access; (6) history of bipolar disorder or positive screen for bipolar disorder based on the Mood Disorder Questionnaire (manic episode can be triggered by BLT); (7) eye disease including glaucoma or retinopathy (BLT contraindications); (8) blindness; (9) night shift work schedules; (10) taking any anti-depressant or anti-anxiety medications; (11) taking other medications that increase sensitivity to light (based on patient self-report).

### Recruitment, consent, enrollment, and randomization

Research staff recruited patients from the pool of patients treated at Columbia University Irving Medical Center/NewYork-Presbyterian Hospital who had experienced an ACS event. Methods to identify patients who experienced recent ACS for study approach included direct referrals from treating providers (both inpatient and outpatient settings), regularly refreshed lists generated from electronic health records, and referrals from other research studies that enrolled ACS patients at our research center. Formal screening for eligibility via questionnaire was done after patients provided informed consent. Individuals who provided verbal consent and screened eligible for the study were enrolled as patients. As this is a pilot study, we used a sample size of N = 15 to enroll enough participants who recently experienced ACS to examine the feasibility of conducting a larger stage II or III RCT of our CC intervention in this patient population.

Patients were randomized in a 2:1 allocation to active CC + SHE treatment or SHE control groups. We used a 2:1 allocation to gain increased experience delivering the CC + SHE intervention in a small sample. A study statistician produced a SAS code to generate a random order of group allocation for 10 CC + SHE treatment patients and 5 SHE control patients. The statistician shared the randomization order with another IRB-approved individual not conducting patient-facing activities in the study, who then placed the group assignments into 15 opaque, sealed envelopes. The randomization assignments remained concealed from both study staff and patients until patients screened eligible for the study. Once a patient screened eligible, study staff opened an envelope, in sequence, to allocate the patient into their study group.

### Interventions

Bright light therapy: During the baseline visit, research coordinators instructed patients randomized to the CC + SHE group to wear a light visor that delivers BLT each morning throughout the 4-week intervention period, starting after awakening and lasting for 30 minutes. BLT was administered via Luminette 3 Light Therapy Glasses, worn like a visor over the eyes. The Luminette 3 contains LEDs emitting a blue-enriched white light reflecting to the retina at ~1,000 lux via a holographic system to ensure correct penetration into the eye without impeding vision. This range of light and intensity is sufficient to synchronize the circadian clock. Patients could engage in other activities (e.g., reading, getting dressed, eating) while the glasses were worn.

Blue light blocking (BLB) lenses: During the baseline visit, research coordinators also instructed patients randomized to the CC + SHE group to wear the BLB lenses in frames (LowBlueLights Zzz Fitover Sleep glasses) each night throughout the 4-week intervention period, starting at 8:00pm and removing the glasses immediately before going to sleep. This time corresponds roughly with the onset of nocturnal melatonin secretion [41], and BLB at this time is hypothesized to encourage physiological mechanisms favoring sleep initiation and circadian stabilization. Staff also instructed patients to wear the glasses during any nocturnal awakenings in which light is turned on, including electronic devices such as smartphones. Polycarbonate lenses of different colors selectively absorb wavelengths of visible light. Depending on the color of lenses used, this results in distinctive filtration of visible wavelengths that are further transmitted along the retinohypothalamic tract. The BLB lenses are orange and filter out short-wavelength light while allowing the other visible spectrum light to pass. The BLB lenses result in a reduction in melanopic irradiance of about 85%. The BLB lenses only make the overall light environment about 30% dimmer. Therefore, the BLB lenses effectively block out most of the short-wavelength/blue light in the visible environment that impacts sleep but does not result in drastic overall dimming/darkening of the light environment. As such, they are unlikely to increase the risk of accidents or falls when used before sleep. We did not specify participants’ timing of sleep and wake during the intervention period.

Sleep hygiene education (SHE): All patients (i.e., in both CC + SHE and SHE control groups) received SHE, which consisted of two brief (~5-minute) videos created by the research team. These videos covered sleep education (basics of sleep and its regulation, impact on health and function) as well as sleep hygiene recommendations (“Sleep Tips”) based on recommendations from the National Heart Lung and Blood Institute and American Academy of Sleep Medicine. Patients who enrolled and screened eligible for the study confirmed viewing the SHE videos before completing the baseline/pre-intervention session. The SHE videos were also transcribed and provided in written form for patients’ reference. Patients in the SHE only control group watched the SHE videos, completed a daily sleep-wake diary, and questionnaires on sleep and mood, but did not use the BLT or BLB devices.

Weekly reminders: As part of assessing adverse events and adherence to the intervention, research staff contacted patients in the CC + SHE intervention group weekly by phone to collect data on side effects and intervention device usage (see below). Accordingly, this phone call acted as a passive weekly reminder to continue using the intervention devices daily. Patients in the control group were also contacted each week to complete a diary of sleep and wake times with research staff.

### Screening tools

We used the Insomnia Symptom Questionnaire (ISQ), a 13-item self-report measure, to screen all patients for the presence of chronic insomnia symptoms (Cronbach α = 0.89) [42]. The ISQ provides a dichotomous outcome (“present” or “absent”) of the definition of insomnia based on the diagnostic criteria from the American Psychiatric Association’s fourth edition of the Diagnostic and Statistical Manual of Mental Disorders (DSM-IV) and is consistent with the American Academy of Sleep Medicine’s Research Diagnostic Criteria. We screened for habitual short sleep duration with a single-item self-report question on how often the individual typically slept 6 hours or less per night over the past month (never, rarely, sometimes, frequently, or always). A reply of frequently (3–4 times per week) or always (5–7 times per week) indicated habitual short sleep duration. Eligibility in the study required a positive screen for chronic insomnia symptoms and/or habitual short sleep duration.

### Primary outcomes

The primary aim of the study was to determine the feasibility of delivering the CC + SHE intervention in individuals who have poor sleep after a recent ACS. We assessed a) feasibility, b) acceptability, c) appropriateness, d) usability, and e) adherence to the 4-week CC + SHE intervention in patients who had ACS with poor sleep (Table 3).

**Table 3:** Summary of study outcomes.


TYPE	NAME	TIME FRAME	BRIEF DESCRIPTION

Primary	Completion: proportion of patients who complete the pilot study	Assessed at conclusion of the study (1-month)	Proportion of patients who completed the 4-week endpoint outcomes assessments

Primary	Feasibility: proportion of patients who report adequate feasibility	Assessed at conclusion of the study (1-month)	Proportion of patients who reported scores ≥4 for their final rating of the intervention’s feasibility (FIM rating)

Primary	Acceptability: proportion of patients who report adequate acceptability	Assessed at conclusion of the study (1-month)	Proportion of patients who reported scores ≥4 for their final rating of the intervention’s acceptability (AIM rating)

Primary	Appropriateness: proportion of patients who report adequate appropriateness	Assessed at conclusion of the study (1-month)	Proportion of patients who reported scores ≥4 for their final rating of the intervention’s appropriateness (IAM rating)

Primary	Usability: proportion of patients who report adequate usability	Assessed at conclusion of the study (1-month)	Proportion of patients who reported scores ≥68 for their final rating of the intervention’s usability (SUS rating)

Primary	Adherence: proportion of patients who adhere to the intervention administration	Assessed continuously from baseline to conclusion of the study (1-month)	Proportion of patients who reported administering the intervention (i.e., morning BLT and evening BLB) on ≥50% (and ≥75%) of the days throughout the 4-week treatment period

Secondary	Insomnia: baseline to 1-month change in insomnia severity	Assessed at baseline and at 1 month	Within-person difference in the Insomnia Severity Index score

Secondary	Sleep quality: baseline to 1-month change in self-report sleep quality	Assessed at baseline and at 1 month	Within-person difference in Pittsburgh Sleep Quality Index global score

Secondary	Sleep duration: baseline to 1-month change in self-report sleep duration	Assessed at baseline and at 1 month	Within-person difference in sleep duration based on Pittsburgh Sleep Quality Index item


AIM: Acceptability of Intervention measure; BLB: Blue light blocking; BLT: Bright light therapy; FIM: Feasibility of Intervention measure; IAM: Intervention Acceptability measure; SUS: System Usability Scale.

We assessed the feasibility, acceptability, and appropriateness of the intervention with valid, reliable, and pragmatic 4-item surveys, including the Feasibility of Intervention Measure (FIM), Acceptability of Intervention Measure (AIM), and Intervention Appropriateness Measure (IAM) [43]. Responses for the FIM, AIM, and IAM ranged from 1: completely disagree to 5: completely agree. Patients also completed the System Usability Scale (SUS) to assess the intervention’s usability [44]. Patients in the CC + SHE group completed these questionnaires at the end of the 4-week study period. We used the following criteria to determine the feasibility, acceptability, appropriateness of the intervention, and usability: 1) the proportion of patients who reported scores ≥4 for their final rating of the intervention’s feasibility (FIM); 2) the proportion of patients who reported scores ≥4 for their final rating of the intervention’s acceptability (AIM); 3) the proportion of patients who reported scores ≥4 for their final rating of the intervention’s appropriateness for improving sleep (IAM); 4) the proportion of patients who reported total scores ≥68 for their final rating of the intervention’s usability (SUS). Completion and adherence were assessed with 1) the proportion of patients who completed the outcome assessments at the study’s conclusion and 2) the proportion of patients who reported administering the intervention. Successful adherence was defined for BLT and BLB separately as using morning BLT and evening BLB on ≥50% (and secondarily, ≥75%) of the days throughout the 4-week treatment period. Adherence to the intervention was assessed via patient self-report. Research staff asked patients to complete a paper-based sleep diary/daily-use log, where they documented the times they administered each of the interventions (i.e., times of starting and stopping BLT, times of donning and doffing BLB glasses, and times of going to bed and waking up) and any notes on usage throughout the 4-week intervention period. Research staff contacted patients weekly to collect the data entered in the sleep diary/daily-use log (in addition to assessing for adverse events).

### Secondary outcomes

As secondary outcomes, we assessed all patients’ insomnia symptom severity, sleep quality, and sleep duration at baseline and the end of the 4-week intervention/study period (Table 3). We assessed sleep outcomes with: 1) pre-post intervention change in insomnia severity (measured as the within-person difference in the total score of Insomnia Severity Index [ISI]; Cronbach α ≥ 0.80) [45,46]; 2) pre-post intervention change in global sleep quality severity measured as the within-person difference in the total score of Pittsburgh Sleep Quality Index (PSQI; Cronbach α ≥ 0.80) [47]; 3) pre-post intervention change in self-reported habitual sleep duration over the prior month, based on the sleep duration item in the PSQI. Scores on the ISI identify the presence and severity of insomnia symptoms, with higher scores indicating worse severity [45]. Scores on the PSQI quantify the degree of global sleep disturbance, with higher scores indicating worse overall sleep quality [47].

### Adverse events

The CC intervention presents minimal known risks to patients. There are no known or anticipated side effects of wearing the BLB glasses as instructed. Known possible side effects of BLT include eyestrain/eye sensitivity, headache, nausea, irritability, and agitation. These side effects are expected to be reversible with cessation of therapy. There is potential that BLT can trigger a manic episode in patients with bipolar disorder. Accordingly, we excluded patients with known diagnoses of bipolar disorder (by patient self-report). We also excluded patients who screened positive for possible bipolar disorder based on the Mood Disorder Questionnaire [48]. A study team member checked in with all patients via brief scheduled calls every week of the 4-week intervention period to ask if the patient was experiencing any side effects related to study devices and followed up with questions to determine the severity of any side effects. To assess for presence of side effects, research staff asked participants: “Over the past week, have you experienced any side effects from either of the treatments? If so, please tell us what you experienced. If possible, please tell us which portion of the intervention (light visor or orange-tinted glasses) you believe may have caused this side effect.”

## Results

### Patients

**Figure 2:**
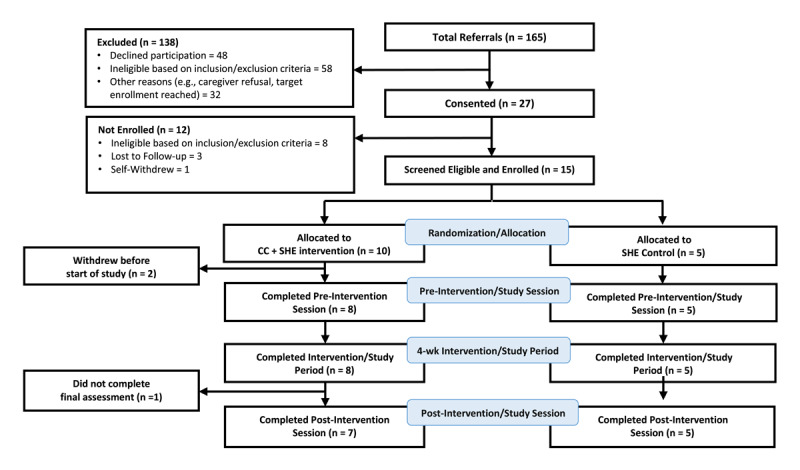
Flow of patients in the study.

The flow of patients through the study is shown in Figure 2. Between October 26, 2022, and January 18, 2024, 165 patients with recent suspected ACS were identified and approached for enrollment. Of these, 138 were excluded because they declined to participate, failed to meet inclusion/exclusion criteria, or for other reasons (e.g., caregiver refusal, target enrollment was reached). Twenty-seven patients consented to the study. Of these, 15 screened eligible and were enrolled in the study. Patients were randomized into CC + SHE intervention (N = 10) or SHE control (N = 5). Two patients from the CC + SHE group withdrew from the study after randomization but before the start of study procedures. One reported being too busy and the other dropped because of personal circumstances. All other patients in the CC + SHE group (N = 8) completed the intervention study period. All SHE control patients (N = 5) completed the study period. For patients in the CC + SHE group who completed the intervention, the mean (standard deviation [SD]) age was 61.8 (10.5) y (range: 43–71 y), 13% were female, 87% were male, 50% were White, 38% Black, 13% Hispanic/Latino. For SHE control, mean (SD) age was 54.4 (17.3) y (range: 32–75 y), 25% were female, 75% were male, 40% were Black, 20% were Asian, and 40% were Hispanic/Latino.

### Primary outcomes

Of the 8 patients who began the CC + SHE intervention, 7 patients (87.5%) completed the entire intervention period and completed outcome assessments at baseline and 4-week endpoint. Of the 8, one patient completed the intervention as a whole but did not complete the outcome assessment at study endpoint. All SHE control patients (100%) completed the tracking period and provided questionnaire data at baseline and 4-week endpoint. Of the 8 patients who completed the CC + SHE intervention period, 7 patients (88%) reported using the BLT ≥50% of the days throughout the intervention period, and 5 patients (63%) reported using the BLT ≥75% of the days. Of the 8 patients who completed the CC + SHE intervention period, all (100%) reported using the BLB ≥50% of the days throughout the intervention period, and 7 patients (88%) reported using the BLT ≥75% of the days.

In patients in the CC + SHE group who underwent the intervention, ratings on the 4-item FIM were ≥4 (indicating sufficient patient-viewed feasibility of the intervention) in 5 patients (71%), with the remaining patients both indicating scores of 3.75 (Table 4). Ratings on the 4-item AIM were ≥4 (indicating sufficient acceptability of the intervention) in 4 patients (57%), with the remaining 3 patients indicating scores of 3, 2.5, and 1. Ratings on the 4-item IAM were ≥4 (indicating patients view the intervention as appropriate to improve sleep) in 2 patients (29%), with the remaining 5 patients indicating scores of 3.75, 3, 3, 1.75, and 1. Ratings on the 10-item System Usability Scale were ≥68 (indicating sufficient usability of the intervention) in all patients (100%).

**Table 4:** Ratings on Feasibility, Acceptability, Appropriateness, and Usability of Intervention.


CC + SHE PATIENT	FIM SCORE	AIM SCORE	IAM SCORE	SUS SCORE

1	3.75	1	1	80

2	5	4.25	3	90

3	4	2.5	1.75	75

4	5	4.25	4.75	97.5

5	3.75	4	3	82.5

6	5	5	5	85

7	4	3	3.75	95

Proportion indicating scores ≥4 (for FIM, AIM, IAM) and ≥68 (for SUS)	71%	57%	29%	100%


Measures reported for intervention group patients with a post-study assessment. FIM: Feasibility of Intervention measure; AIM: Acceptability of Intervention measure; IAM: Intervention Acceptability measure; SUS: System Usability Scale.

### Secondary outcomes

Individual changes in scores on the ISI, PSQI global score, and self-reported sleep duration from baseline to 4-week follow-up are shown in Table 5. Improvement (i.e., reduction) in ISI was seen in 71% of patients in the CC + SHE group and 40% of SHE controls (mean change: –5.7 vs. –3, Cohen’s d = 0.35). Improvement (i.e., reduction) in PSQI global score was seen in 71% of CC + SHE patients and 80% of SHE controls (mean change: –3.6 vs. –3.2, Cohen’s d = 0.10). Increases in self-reported sleep duration were seen in 71% of patients in the CC + SHE group and 60% of SHE controls (mean change: 1.0 vs. 0.3 hours, Cohen’s d = 0.53).

**Table 5:** Change (pre-post) in sleep metrics for CC + SHE intervention (N = 7) and SHE control (N = 5) patients.


ISI CHANGE	CC + SHE	SHE	PSQI CHANGE	CC + SHE	SHE	SLEEP DURATION CHANGE	CC + SHE	SHE

	3	–7		1	–9		–0.5	2.5

	–1	0		–3	0		1	–1

	–4	0		0	–1		0	1

	–1	–9		–6	–4		2.5	0.5

	–20	1		–5	–2		1.5	–1.5

	–18	–		–10	–		2	–

	1	–		–2	–		0.5	–

**Mean (SD)**	–5.7 (9.3)	–3.0 (4.6)		–3.6 (3.8)	–3.2 (3.6)		1.0 (1.1)	0.3 (1.6)

**Cohen’s d**	0.35			0.10			0.53	


Values are shown for individual patients, as well as group means and Cohen’s d for comparisons between groups. CC + SHE: Combined chronotherapy + SHE intervention; SHE: Sleep hygiene education control; ISI: Insomnia Severity Index; PSQI: Pittsburgh Sleep Quality Index; SD: Standard deviation.

### Adverse events

Two patients in the CC + SHE group reported side effects potentially related to the BLT (Table 6). One experienced a headache during the first week of the BLT (an expected side effect), which was reported as mild. The patient stopped using the BLT for 3 days, and the headache was resolved. The patient resumed the BLT for the remaining 3 weeks of the intervention period. Another patient reported experiencing headaches, eye sensitivity, and eye pain in response to BLT (expected side effects) at the start of the intervention period. The patient decided to stop using the BLT but continued to use the BLB for the remainder of the 4-week intervention period.

**Table 6:** Reported side effects for CC + SHE intervention (N = 8) and SHE control (N = 5) patients.


SIDE EFFECT	CC + SHE	SHE

Eyestrain/eye sensitivity, n	1	0

Headache, n	2	0

Nausea, n	0	0

Irritability, n	0	0

Agitation, n	0	0

Other, n	0	0


CC + SHE: Combined chronotherapy + SHE intervention; SHE: Sleep hygiene education control.

## Discussion

Here, we describe the rationale, design, protocol, and initial pilot testing of a light-focused CC intervention targeting disturbed sleep following ACS. In a pilot RCT, we found high adherence to the CC + SHE intervention, consisting of morning bright light exposure and evening short-wavelength light avoidance, among those who initiated the treatment, and high ratings of patient-perceived feasibility and usability in a small group of individuals with insomnia symptoms and/or short sleep duration following ACS. We also examined the preliminary effects of the CC + SHE intervention and found that insomnia symptoms, sleep quality, and sleep duration were improved in 71% of patients. This suggests that it is plausible that the minimal risk intervention could result in clinically significant benefits in ACS patients. The trial also demonstrated feasibility of recruitment and retention in the RCT protocol among intervention and control group patients. Our next step is to conduct a larger stage II efficacy RCT trial to more definitively understand whether this approach can improve sleep in ACS patients. We plan to develop this non-pharmacologic approach to improving sleep in this patient population further with larger-scale efficacy trials since sleep may be a secondary risk prevention target in individuals who have experienced a cardiac event. Future work can examine whether this intervention improves markers of CVD risk (e.g., blood pressure or inflammatory markers).

As described above, this protocol was developed as a Stage IA-IB feasibility and pilot study aiming to refine, modify, and adapt existing interventions [40]. Primarily designed to test feasibility, this study includes several important limitations. First, we assessed patient adherence to the intervention components only with self-report measures (device use log). Future work should develop methods to objectively monitor device use as a measure of intervention fidelity. Potential approaches to track the use of the Luminette/BLT can include a built-in monitor to determine when the device is turned on and off. Times of wearing the BLB glasses can be confirmed by affixing a skin temperature sensor to the glasses arm or hinge. Second, the study did not include biomarkers of circadian rhythms. This is important because the intervention is designed to improve sleep, at least in part, via the circadian system. Future work should include measures of the circadian system, including phase and amplitude, to better determine physiologic mechanisms of action. Third, patients were included if they had either insomnia symptoms and/or short sleep duration. While insomnia and short sleep duration can frequently co-occur and have important health ramifications [49], they are distinct constructs that can have different implications on both physiology and treatment approaches. We chose to include both here because a) we were primarily assessing the feasibility of administering the intervention in a sample with sleep disorders, and b) the interventions have been shown to improve both sleep duration and sleep quality/insomnia symptoms [38,39].

An important point about the intervention is that by focusing on light exposure patterns in the morning and pre-sleep period, the intervention aims to improve sleep in part by improving circadian function, boosting circadian amplitude, and ensuring that the body’s biological rhythm is synchronized to the day-night solar cycle. However, not all insomnia and short sleep duration are caused by circadian disturbance. Other methods not tested here (e.g., CBT-I or behavioral sleep extension) may be better suited for some individuals to improve sleep. The development of the CC intervention for poor sleep in individuals who have experienced ACS points to the potential for more precision medicine approaches in which interventions can be aligned with etiologies of poor and/or inadequate sleep.

Despite these limitations, the study includes several strengths and represents an important next step in the development of non-pharmacologic treatments for sleep disturbances in clinical populations. All study procedures, including intervention administration and outcomes assessment, were conducted remotely. This increased the ease of study procedures by saving patients trips to the laboratory. The intervention was self-administered by patients. Self-administration of the intervention helps overcome potential obstacles some individuals face in obtaining therapist-delivered treatments for sleep disturbance. The intervention was easy to administer as verified by patients’ high feasibility ratings, noninvasive, and relatively inexpensive and safe. This is particularly important as the target population is patients recovering from a cardiac event who often wish to avoid pharmacotherapy or inconvenient intervention visits. This intervention approach, which utilized wearable devices, is also adaptable and can potentially improve sleep in various patients and settings, including the inpatient hospital setting, where environmental factors are known to be severely disruptive to circadian rhythms and sleep. For instance, dynamic hospital lighting systems which enhance bright light in the daytime and deplete short wavelength and bright light in the evening, may be an effective way to counteract the adverse effects of overly dim daytime light and evening and nighttime bright light on patient recovery [50]. However, many of the dynamic or circadian lighting systems in hospitals studied to date require structural changes to the lighting apparatus. The approach proposed here, using wearable devices for BLT and BLB, can be a way to achieve these effects in an affordable, scalable way.

While there was high completion of the study by patients in the intervention and control groups, high (self-reported) adherence to the intervention, and patients generally reported the intervention as usable, relatively fewer patients perceived the intervention as appropriate to improve sleep, even though the majority of patients in the CC + SHE group had ISI and PSQI scores that improved, and self-reported sleep duration increased. Light exposure patterns are touched on, albeit briefly, in the sleep hygiene education program. Specifically, patients were informed of the importance of avoiding exposure to too much bright light in the evening before sleep, and getting a high amount of bright light during the day was recommended to promote optimal sleep. Going beyond standard sleep hygiene education by further highlighting the mechanistic pathways by which light in the evening can disturb sleep and daytime light can improve sleep may enhance patients’ understanding of the intervention approach. Increasing patient-perceived appropriateness of the intervention may also bolster intervention acceptability and adherence. A run-in phase may be needed to ensure interest in participation prior to randomization to prevent drop-out in a larger trial.

Here, we described the rationale, protocol, and preliminary testing in an RCT of a “combined chronotherapy” intervention, consisting of morning BLT and evening short-wavelength light avoidance (BLB), which aims to alleviate circadian desynchrony and improve sleep by augmenting helpful daytime light and reducing sleep/circadian-disturbing short-wavelength light exposure at night. Preliminary findings show this non-pharmacologic chronotherapeutic intervention to be feasible as a sleep-focused therapy in patients who experienced ACS. Furthermore, we showed that it is feasibility to conduct an RCT comparing CC + SHE vs. SHE, alone. Data collected in this pilot study will help guide efficacy testing of the proposed CC intervention in a larger Stage II trial. If successful, this non-pharmacologic approach has broad applicability to a wide range of patient groups, is scalable, and is easy to implement in almost any clinical or at-home setting.

## Data Accessibility Statement

The datasets used and/or analyzed during the current study are available from the corresponding author on reasonable request.

## Additional File

The additional file for this article can be found as follows:

10.5334/jcr.250.s1 Supplementary File. SleepWell_CONSORT Checklist.

## Abbreviations

ACS: acute coronary syndrome

AIM: Acceptability of Intervention Measure

BLB: blue light blocking

BLT: bright light therapy

CBT-I: Cognitive behavioral therapy for insomnia

CC: combined chronotherapy

CVD: cardiovascular disease

FIM: Feasibility of Intervention Measure

IAM: Intervention Appropriateness Measure

ISI: Insomnia Severity Index

LED: light-emitting diode

PSQI: Pittsburgh Sleep Quality Index

SHE: sleep hygiene education

SUS: System Usability Scale
